# Isolated IgG4-related cholecystitis with localized gallbladder wall thickening mimicking gallbladder cancer: a case report and literature review

**DOI:** 10.1186/s12876-022-02179-z

**Published:** 2022-03-04

**Authors:** Yuko Harada, Kisyo Mihara, Ryusuke Amemiya, Masashi Nakagawa, Ryota Hanada, Kentaro Inoue, Masaya Shito, Hideki Orikasa, Koichi Aiura

**Affiliations:** 1 Department of Surgery, Kawasaki Municipal Hospital, Shinkawadori 12-1, Kaswasaki-ku, Kawasaki-shi, Kanagawa 210-0013 Japan; 2 Department of Internal Medicine, Kawasaki Municipal Hospital, Shinkawadori 12-1, Kaswasaki-ku, Kawasaki-shi, Kanagawa 210-0013 Japan; 3 Department of Pathology, Kawasaki Municipal Hospital, Shinkawadori 12-1, Kaswasaki-ku, Kawasaki-shi, Kanagawa 210-0013 Japan; 4 Center for Endoscopy, Kawasaki Municipal Hospital, Shinkawadori 12-1, Kaswasaki-ku, Kawasaki-shi, Kanagawa 210-0013 Japan

**Keywords:** IgG4-related cholecystitis, Gallbladder wall thickening, Gallbladder cancer, Case report

## Abstract

**Background:**

IgG4-related cholecystitis, which is a manifestation of IgG4-related disease in the gallbladder, is associated with autoimmune pancreatitis or IgG4-related sclerosing cholangitis in most cases; isolated gallbladder lesions without systemic manifestations are very rare. Gallbladder wall thickening is often diffuse, but sometimes localized, in which case, differentiation from gallbladder cancer becomes difficult. The characteristic features of IgG4-related cholecystitis on imaging that would enable differentiation from gallbladder cancer remain poorly described.

**Case presentation:**

We present a rare case of isolated IgG4-related cholecystitis with localized gallbladder wall thickening that was clinically difficult to distinguish from malignancy before resection. An 82-year-old man was referred to our hospital because of gallbladder wall thickening on abdominal ultrasonography without any symptoms. Dynamic computed tomography of the abdomen showed localized wall thickening from the body to the fundus of the gallbladder that was enhanced from an early stage with a prolonged contrast effect. There were no other findings, such as pancreatic enlargement and bile duct dilatation. Magnetic resonance cholangiopancreatography revealed neither dilatation nor stenosis of the bile duct and pancreatic duct. Endoscopic ultrasonography (EUS) showed a smooth layered thickening of the gallbladder wall with a maximum thickness of 6 mm and a well-preserved outermost hyperechoic layer in the same area. Laparoscopic cholecystectomy was performed because malignancy could not be completely ruled out. Pathological examination of a resected specimen revealed IgG4-positive plasma cell infiltration, fibrosis, and phlebitis. Although the serum IgG4 level measured after resection was normal, the condition was ultimately diagnosed as probable IgG4-related cholecystitis according to the 2020 revised comprehensive diagnostic criteria for IgG4-related disease. The EUS images reflected the pathological findings, in which lymphocytic infiltration was distributed in a laminar fashion in the gallbladder wall.

**Conclusions:**

Although rare, isolated IgG4-related cholecystitis with localized wall thickening mimicking gallbladder cancer remains a clinical problem. A smooth laminar thickening of the gallbladder wall on EUS imaging could be one of the most informative characteristics for differentiating IgG4-related cholecystitis from gallbladder cancer.

## Background

IgG4-related disease is an immune-mediated fibroinflammatory condition characterized by frequent elevation of serum IgG4 levels, multiple organ involvement with abundant lymphocytic infiltration containing an accumulation of IgG4-positive plasma cells, and fibrosis [1]. In general, IgG4-related cholecystitis, which is a manifestation of IgG4-related disease in the gallbladder, is associated with IgG4-related diseases, such as autoimmune pancreatitis or IgG4-related sclerosing cholangitis, and presents with diffuse, circumferential thickening of the gallbladder wall in most cases [2–4]. However, a localized thickening type of IgG4-related cholecystitis sometimes exists [5–7]. Isolated IgG4-related cholecystitis is a very rare disease [8, 9] that is often difficult to distinguish from malignancy, especially localized IgG4-related cholecystitis mimicking gallbladder cancer. In those cases, surgical resection is often performed, because it is difficult to obtain findings negative for cancer before resection. The characteristic features of IgG4-related cholecystitis on imaging remain poorly described.

In this article, we report a rare case of isolated IgG4-related cholecystitis with an interesting appearance on endoscopic ultrasonography (EUS), along with a review of the literature.

## Case presentation

**Fig. 1:**
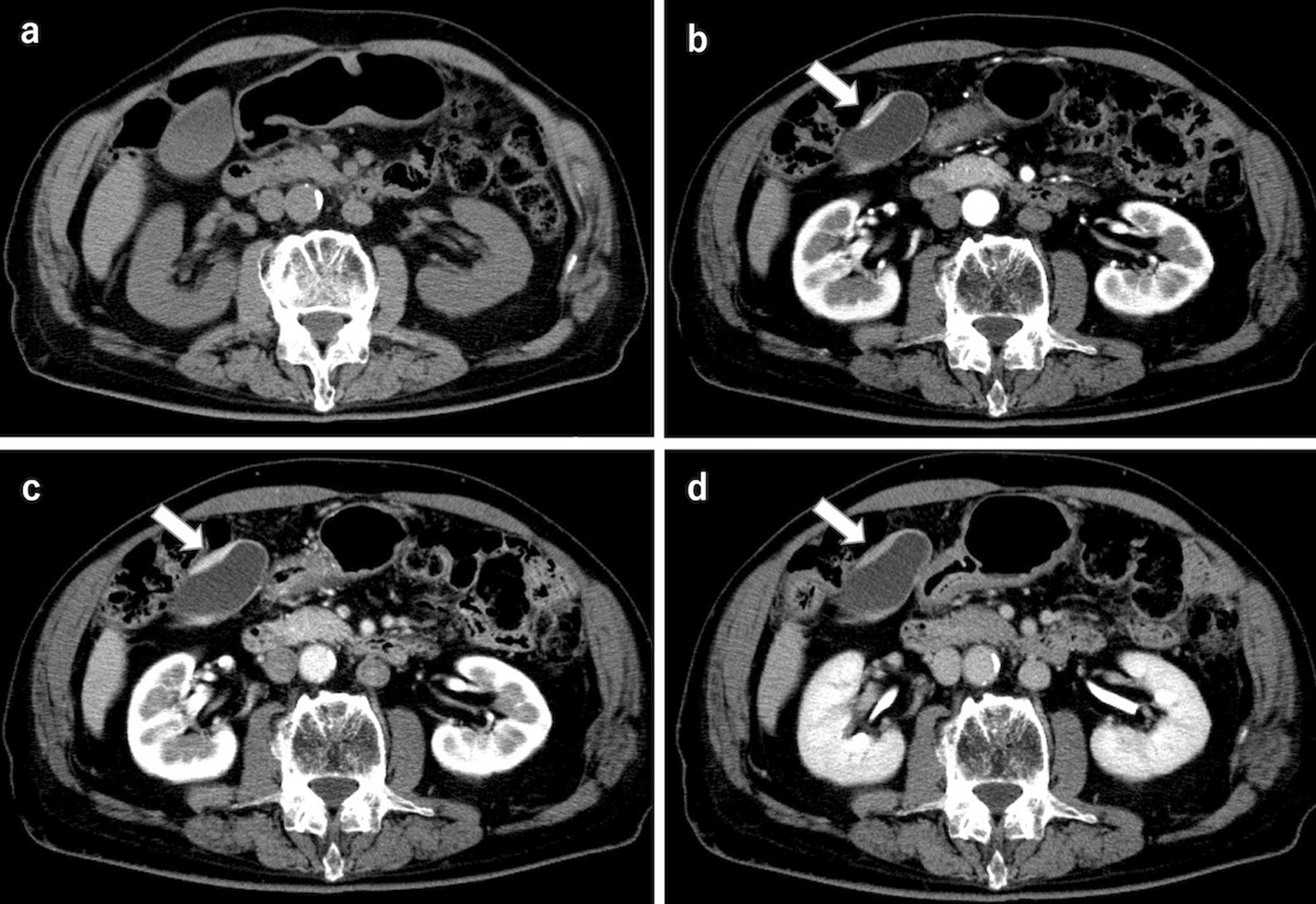
Dynamic CT shows localized wall thickening (**a**; plain scan) with early staining (**b**; arterial phase) and a prolonged contrast effect (**c**; portal phase**, d**; equilibrium phase) from the body to the fundus of the gallbladder (arrows), but no other findings, such as pancreatic enlargement

**Fig. 2:**
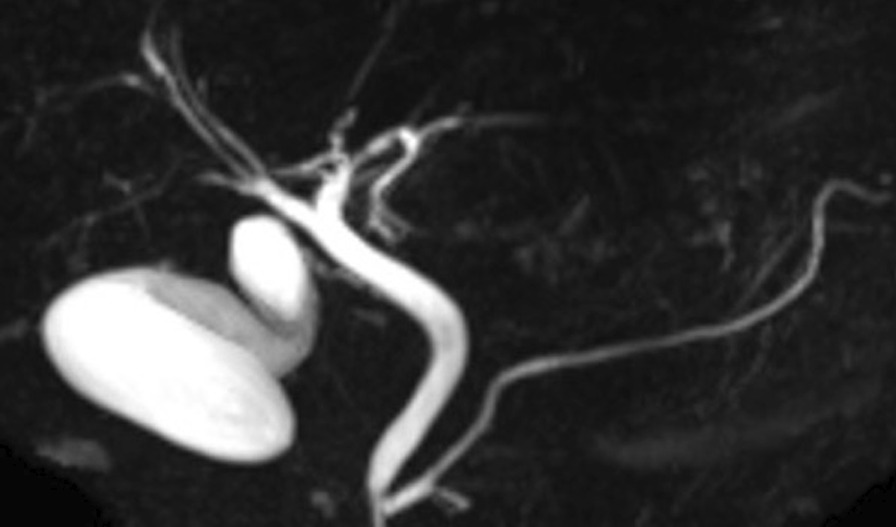
MRCP shows no obvious abnormal findings, such as narrowing of the bile duct or pancreatic duct

**Fig. 3:**
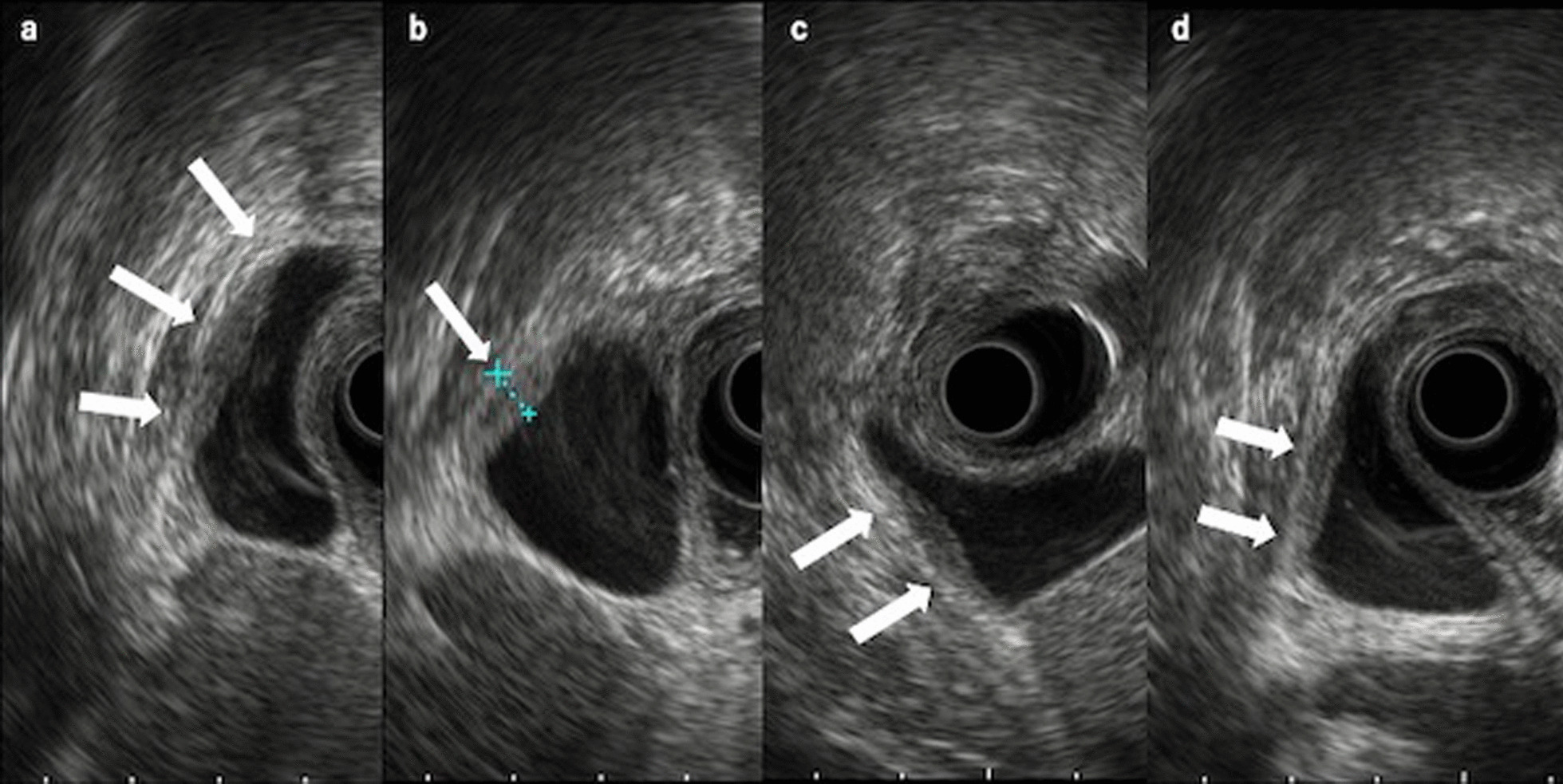
EUS findings of scans from various angles (**a-d**) show localized smooth wall thickening at the fundus of the gallbladder (arrows), with a maximum thickness of 6 mm. The interior is depicted in layers, with the outermost hyperechoic layer of the same area well preserved

**Fig. 4:**
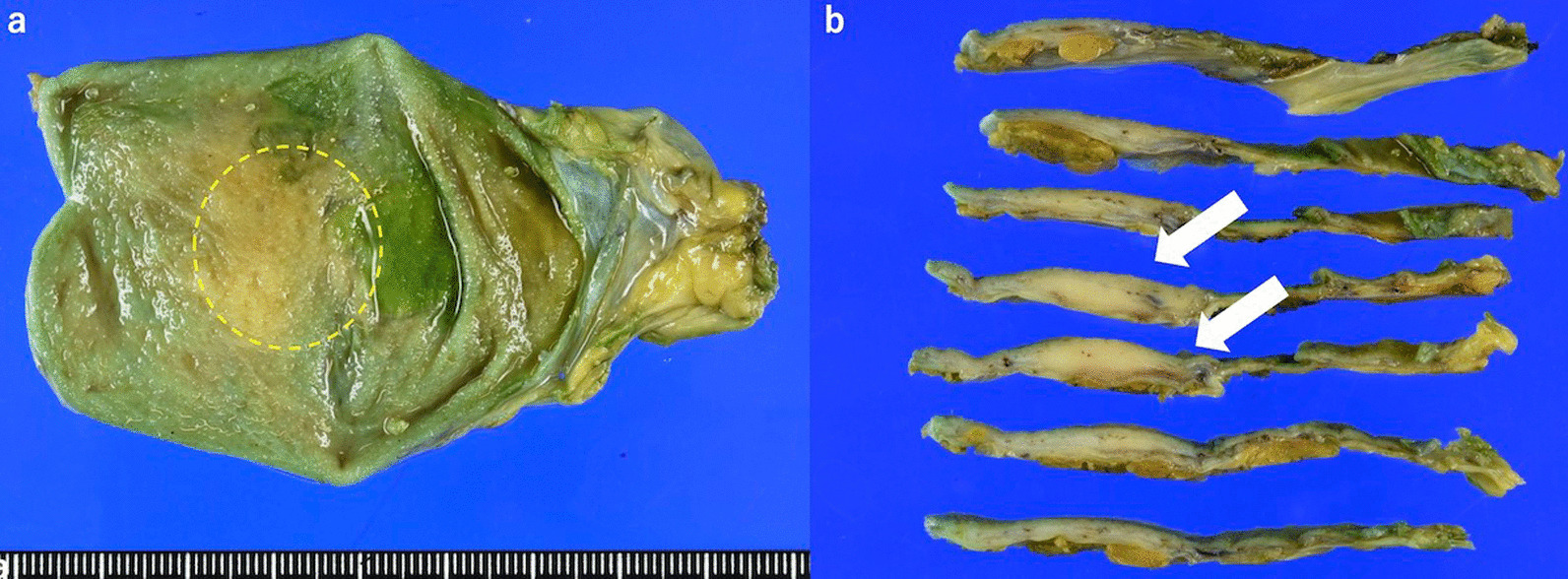
Resected specimen of the gallbladder. **a** 20 × 20 mm-sized induration in the center of the body (dashed circle). **b** A grayish-white, full-thickness wall thickening is observed on the cut surface (arrows)

**Fig. 5:**
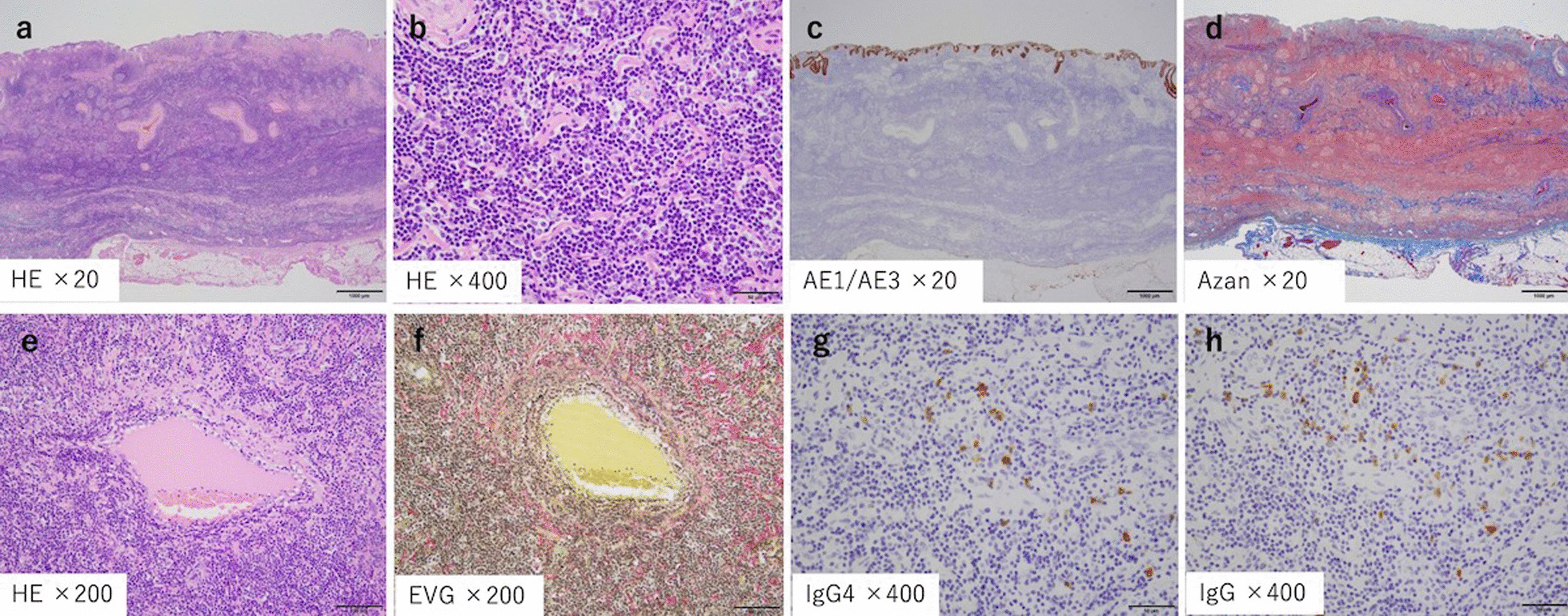
Pathologic findings. **a**, **b** Hematoxylin–Eosin stain showing a high-grade lymphocytic and plasma cell infiltration and fibrosis in all layers without malignant findings. **c** Immunohistochemical staining of cytokeratin AE1/AE3 showing that the mucosal epithelium of the gallbladder remained normal with no evidence of destruction by lymphocytic infiltration. **d** Azan stain showing that lymphocytic infiltration was distributed in a laminar fashion in the gallbladder wall. **e, f** Although there was no obstruction, there was an apparent phlebitis (e: Hematoxylin–Eosin stain, f: EVG stain). **g, h** More than 10 IgG4-positive cells were observed per high-power field (g: IgG4 immunohistochemical stain), and the IgG4/IgG positive cell ratio exceeded 40% (h: IgG immunohistochemical stain)

An 82-year-old man presented to the local clinic with abnormal liver function, and gallbladder wall thickening was found on abdominal ultrasonography (US). He was referred to our hospital for further examination. At the time of the initial visit, a physical exam revealed no abnormalities in vital signs and no abdominal findings. The patient had a 12-year history of hyperlipidemia and had undergone unilateral lobectomy plus isthmectomy for follicular carcinoma of the right thyroid gland in his 70 s. On admission, a complete blood count and serum chemical studies reflected no abnormal findings, no increase in inflammatory response, and tumor markers within normal limits (carcinoembryonic antigen 1.2 ng/mL, carbohydrate antigen 19–9 9.0 U/mL). Dynamic computed tomography (CT) showed localized wall thickening from the body to the fundus of the gallbladder, which was enhanced from an early stage and showed a prolonged contrast effect. There were no other findings, such as pancreatic enlargement, bile duct dilatation, or retroperitoneal fibrosis (Fig. 1). Magnetic resonance imaging (MRI) showed gallbladder wall thickening in the gallbladder body on T2-weighted images and a high signal on diffusion-weighted images. Contrast-enhanced dynamic MRI showed wall thickening with early enhancement, as seen on CT, and no intramural cyst-like structures suggestive of Rokitansky-Aschoff sinuses (RAS). Magnetic resonance cholangiopancreatography (MRCP) revealed no dilatation, stenosis, or border irregularity of the bile duct and pancreatic duct (Fig. 2). EUS showed a localized, smooth wall thickening at the base of the gallbladder with a maximum thickness of 6 mm, and the interior was delineated in a laminar fashion, while the outermost hyperechoic layer in the same area was well preserved (Fig. 3). Although the possibility of inflammatory disease, such as chronic cholecystitis, was considered, malignancy could not be completely ruled out, the patient underwent laparoscopic whole layer cholecystectomy for the diagnosis and treatment. No malignant findings were reported in the intraoperative rapid diagnostic test of the gallbladder, and the surgery was completed. A resected specimen of the gallbladder showed a 20 × 20 mm-sized induration in the center of the body, and a grayish-white, full-thickness wall thickening was observed on the cut surface (Fig. 4). Pathological examination revealed a high-grade lymphocytic and plasma cell infiltration and fibrosis in all layers without malignant findings (Fig. 5a, b). The mucosal epithelium of the gallbladder remained normal, with no evidence of destruction by lymphocytic infiltration (Fig. 5c), and lymphocytic infiltration was distributed in a laminar fashion in the gallbladder wall (Fig. 5d). Although there was no obstruction, there was an apparent phlebitis (Fig. 5e, f). In addition, more than 10 IgG4-positive cells were observed per high-power field (HPF) (Fig. 5g), and the IgG4/IgG positive cell ratio exceeded 40% (Fig. 5h). The pathology findings of the cystic duct wall of the gallbladder showed only mild chronic inflammatory cell infiltration, including a small number of plasma cells without IgG4-positive cells, suggesting no involvement of IgG4-related disease. According to the 2020 revised comprehensive diagnostic criteria for IgG4-related disease [10], serum IgG4 levels after surgery were normal in serological diagnosis [item 2], but the other two items providing clinical and radiological features [item 1] and pathological diagnosis [item 3] (> 10 IgG4 + cells/HPF and IgG4 + /IgG cell ratio > 40%) were relevant, and thus the final diagnosis was probable IgG4-related cholecystitis. The patient was followed up with laboratory tests and CT scans from the neck to the abdomen and has remained free of relapse without steroid administration for one and a half year after surgery.

## Discussion and conclusions

Herein, we report the rare case of a patient with localized IgG4-related cholecystitis without any other organ involvement, including the pancreas and bile duct. Interestingly, EUS findings of the gallbladder wall thickening revealed a characteristic feature, that is, a smooth layered thickening, which could be useful for differentiating IgG4-related cholecystitis from gallbladder cancer on imaging.

IgG4-related cholecystitis is considered a lesion of IgG4-related disease. It usually presents as a gallbladder wall thickening synchronous with autoimmune pancreatitis or IgG4-related sclerosing cholangitis [3, 11, 12], and isolated gallbladder lesions are very rare [8, 9]. The thickening of the gallbladder wall is often diffuse [2–4], but it is sometimes localized, in which case, differentiation from gallbladder cancer becomes problematic [4, 6]. Even in the presence of autoimmune pancreatitis or IgG4-associated sclerosing cholangitis, it is necessary to exclude gallbladder carcinoma, and as a result, many cases are diagnosed only after overly invasive surgical resection.

The pathological features are characterized by a dense lymphoplasmacytic infiltration, IgG4-positive cell infiltration, inflammation often involving all layers, storiform fibrosis, obstructive phlebitis, and normal mucosal epithelium of the gallbladder [10, 13].

A PubMed search for past case reports from January 2005 to August 2021 using the search term "IgG4-related cholecystitis" retrieved 16 cases (Table 1) [5–9, 16–24]. Six of 17 cases showed diffuse wall thickening, while the remaining 11 cases (including our case) presented with localized thickening. Preoperative serum IgG4 levels in patients with IgG4-related cholecystitis were increased in 7 cases. Only 4 cases of so-called "isolated" gallbladder disease without other organ involvement, including autoimmune pancreatitis and sclerosing cholangitis, were found in previously reported 16 cases and our case report will be the 5th reported case. Ten cases underwent radical cholecystectomy with liver resection tailored toward presumed malignant neoplasms.

**Table 1:** Previously reported cases of IgG4-related cholecystitis

No	References	Age	Sex	Isolated	Type	AUS	EUS	Dynamic CT early enhancement	Operation	Preoperative IgG4 (mg/dl)	Postoperative IgG4 (mg/dl)
1	Gumbs et al. [16]	68	F	−	Local	−	−	+	RC	NA	WNL
2	Matsubayashi et al. [17]	62	M	−	Diffuse	+	−	−	–	764	NA
3	Kawakami et al. [7]	55	M	−	Local	+	−	+	RC	455	NA
4	Leise et al. [18]	76	M	−	Diffuse	+	−	NA	–	WNL	NA
5	Lee et al. [19]	59	M	−	Local	−	−	−	–	WNL	NA
6	Shin et al. [9]	58	M	+	Diffuse	−	−	−	RC	NA	NA
7	Feely et al. [20]	61	F	−	Local	−	−	NA	RC	NA	NA
8	Feely et al. [20]	71	F	+	Local	−	−	NA	RC	NA	WNL
9	Feely et al. [20]	53	M	−	Local	−	−	NA	RC	NA	NA
10	Inoue et al. [5]	60	F	−	Local	+	−	+	RC	813	NA
11	Li et al. [21]	61	M	−	Diffuse	−	−	+	−	1750	1560
12	Takahashi et al. [8]	18	M	−	Local	−	−	−	RC	WNL	NA
13	Ishigamiet al. [22]	82	M	−	Diffuse	+	−	−	SC	943	NA
14	Kulkarni et al. [23]	48	M	−	Local	+	−	−	RC	NA	WNL
15	Ichinokawa et al. [6]	56	M	+	Local	+	−	−	RC	721	303
16	Jearth et al. [24]	57	M	+	Diffuse	+	−	NA	SC	2610	NA
17	Our case	82	M	+	Local	+	+	+	SC	NA	WNL

AUS, abdominal ultrasonography; EUS, endoscopic ultrasonography; Isolated, isolated cholecystitis without other organ involvement; NA, not assessed; RC, radical cholecystectomy with liver resection; SC, simple cholecystectomy without liver resection; WNL, within normal limit

Localized thickening was more common than diffuse thickening in the previously published case reports. This may be because localized thickening of the gallbladder wall is more difficult to differentiate from gallbladder cancer. Chronic cholecystitis, xanthogranulomatous cholecystitis, and gallbladder carcinoma are among the differential diagnoses of thickened gallbladder wall lesions, but distinguishment from gallbladder carcinoma is of utmost importance. US, CT, MRI, positron emission tomography (PET)/CT, and EUS are commonly used to evaluate IgG4-related disease. PET/CT imaging is considered a useful tool for the differential diagnosis of IgG4-related disease from malignant tumors. However, the utility of serial fluorodeoxyglucose-PET studies has not been shown in guiding treatment decisions [14]. There are no definite opinions on the characteristic imaging findings of IgG4-related cholecystitis, and it is still accepted that it is difficult to distinguish it from gallbladder cancer on diagnostic imaging. Recently, however, Zhang and colleagues compared CT and MRI images of gallbladder cancer and IgG4-related cholecystitis and reported that thickening of the gallbladder wall in a layered pattern and RAS changes in the gallbladder wall are useful for differentiating the two [4]. Furthermore, patients with gallbladder cancer had lower enhancement of the nodules in the portal phase, which was not observed in patients with IgG4-related cholecystitis [4]. In our case and previously reported 4 cases (Table 1), gallbladder wall thickening was strongly enhanced from an early phase and showed a prolonged contrast until the delayed phase on dynamic CT, suggesting the possibility of malignancy. In this study, only EUS showed a finding inconsistent with cancer. Thus, we compared the EUS images of our patient with the pathological findings. A smooth layered thickening in the EUS (Fig. 3) reflected histological layered lymphocytic infiltration in the gallbladder wall (Fig. 5d). A diagnostic EUS was not performed in the past reported 16 cases (Table 1). Patients with such appearance on EUS should be considered for differential diagnosis, and serum IgG4 levels, as well as other clinical indicators, should be assessed. This is because elevated serum IgG4 levels are considered to provide an informative standard for preoperative diagnosis of IgG4-related diseases, but it has been reported that serum IgG4 levels are normal in 3% to 30% of patients [14]. In our case, the serum IgG4 level obtained after resection was normal. IgG4-related cholecystitis has been reported to respond well to steroids [15]. Therefore, if IgG4-related cholecystitis is suspected, a diagnostic steroid trial or a diagnostic laparoscopic cholecystectomy may be considered instead of overly invasive surgery for malignancy.

The findings of this case report are limited because the EUS findings are of only one case with IgG4-related cholecystitis. In addition, we did not measure the levels of serum IgG4 before surgery. Furthermore, we have been following this patient to determine whether ectopic relapse occurred in other organs after surgery for only 1.5 years. We must accumulate more cases with IgG4-related cholecystitis to elucidate the clinical characteristics and typical features on imaging, especially EUS, of IgG4-related cholecystitis and to establish a differential diagnosis from gallbladder cancer.

In this study, we experienced a rare case of localized IgG4-related cholecystitis without other organ involvement, such as autoimmune pancreatitis or IgG4-related sclerosing cholangitis. The finding of smooth, laminar wall thickening of the gallbladder wall by EUS may be a key point differentiating this disease from gallbladder carcinoma.

## Data Availability

Not applicable.

## Abbreviations

EUS: Endoscopic ultrasonography
US: Ultrasonography
CT: Computed tomography
MRI: Magnetic resonance imaging
RAS: Rokitansky-Aschoff sinuses
MRCP: Magnetic resonance cholangiopancreatography
HPF: High-power field
PET: Positron emission tomography
